# CADASIL: Migraine, Encephalopathy, Stroke and Their Inter-Relationships

**DOI:** 10.1371/journal.pone.0157613

**Published:** 2016-06-16

**Authors:** Rhea Yan Ying Tan, Hugh Stephen Markus

**Affiliations:** Stroke Research Group, Department of Clinical Neurosciences, University of Cambridge, Cambridge, United Kingdom; Taipei Veterans General Hospital, TAIWAN

## Abstract

**Background:**

Migraine is common in Cerebral Autosomal Dominant Arteriopathy with Subcortical Infarcts and Leukoencephalopathy (CADASIL) but its treatment responses are not well described, and its relationship to stroke risk unknown. Encephalopathy is a less common presentation; it has been suggested it is related to migraine. We characterised migraine patterns and treatment responses in CADASIL, and examined associations between migraine and both stroke risk and encephalopathy.

**Methods:**

300 symptomatic CADASIL patients were prospectively recruited from a national referral clinic over a nineteen year period, from 1996 to 2015. Data was collected using a standardised questionnaire. Migraine was classified according to the International Classification of Headache Disorders, 3^rd^ edition (beta version). A cross-sectional analysis was carried out on the data collected.

**Results:**

Migraine was present in 226 (75.3%), and the presenting feature in 203 (67.7%). It was usually accompanied by aura (89.8%). Patients showed variable responses to a variety of drugs for migraine. Of 24 given triptans, 45.5% had consistent or partial responses. None had complications following triptans. Thirty-three (11.0%) patients experienced encephalopathy lasting on average 8.1 ± 3.4 days. Patients with migraine with aura had higher odds of encephalopathy (OR = 5.4; 95%CI 1.6–28.4; p = 0.002). Patients with confusional aura had higher odds of encephalopathy than those with other aura types (OR = 2.5, 95%CI = 1.0–5.8, p = 0.04). There was also no increase in risk of encephalopathy with sex or age at onset of migraine. Migraineurs had a lower stroke risk than non-migraineurs (HR = 0.46, 95%CI 0.3–0.6, p = 2.1x10^-6^).

**Conclusions:**

Migraine with aura is a prominent feature of CADASIL. Treatment responses are similar to those seen in the general migraine population and no complications were observed with triptans. Migraine with aura was associated with increased risk of encephalopathy suggesting they may share pathophysiological mechanisms. There was no increased stroke risk associated with migraine, but risk appeared to be reduced although this finding needs confirming.

## Introduction

Cerebral Autosomal Dominant Arteriopathy with Subcortical Infarcts and Leukoencephalopathy (CADASIL) is the most common monogenic form of stroke, and is caused by mutations in the *NOTCH3* gene.[1] Although pathological changes can be seen in both systemic and cerebral vessels, clinical features of CADASIL are confined to the central nervous system, causing subcortical lacunar infarcts and cognitive impairment progressing to vascular dementia.[2]

Migraines are often the earliest feature of disease and have been reported in up to 75% of cases.[2–4] The pattern of migraines differs from that seen in the general population with a predominance of migraine with aura, and frequent reports of complicated and prolonged aura.[2]

Despite its prevalence there have been few detailed studies of migraine in CADASIL.[5–8] Furthermore, little is known about how it responds to therapy and whether the management of migraine in CADASIL should differ from that of migraine in the general population.[9–12] Epidemiological studies have demonstrated that migraine, in particular migraine with aura, is an independent risk factor for stroke in the general population.[13–16] However, whether the presence of migraine with aura in CADASIL is predictive of an earlier onset of stroke and a more severe phenotype is unknown. Another related clinical phenotype of CADASIL is encephalopathy or “CADASIL coma”. This can be preceded by an acute migraine headache,[2,17] but the associations between migraine and encephalopathy are poorly understood.

In a large cohort of patients with CADASIL we determined the prevalence and characteristics of migraine. We recorded responses to migraine medication, and analysed the relationship between migraine, and both stroke and encephalopathy.

## Methods

Data from 300 symptomatic CADASIL patients (170 females, 130 males) seen in a UK CADASIL National Referral Service was collected prospectively over a nineteen year period, from 1996 to 2015. All patients had a confirmed diagnosis of CADASIL, either by direct sequencing of the *NOTCH3* gene to identify a pathogenic cysteine-changing mutation (n = 296), by electron microscopy of a skin biopsy demonstrating granular osmiophilic material (n = 2) or by characteristic imaging features of CADASIL, with a family history of genetically-confirmed CADASIL (n = 2). Patients diagnosed with CADASIL on pre-symptomatic genetic testing were excluded from this study. Patients were evaluated and examined by consultant neurologists, written and informed consent was obtained, and data was collected using a standardised questionnaire at first review and at follow-up encounters.

Patients were diagnosed with migraine at the point of care according to the International Classification of Headache Disorders (ICHD). The first edition (ICHD-1) was used from 1996 to 2004, followed by ICHD-2 from 2004 to 2013, and ICHD-3 (beta version)[18] from 2013 to 2015. Details of migraine episodes such as the duration, frequency and auras were collected.

A cross-sectional analysis was carried out in 2015. The data was reviewed and all episodes of migraine were classified according to the ICHD-3 beta, where migraine with aura was defined as episodes where the ‘aura is accompanied, or followed within 60 minutes, by headache’. Aura types were classified according to typical aura (visual, sensory and/or speech or language symptoms with no motor weakness or monocular field defect), hemiplegic (visual, sensory and/or speech or language symptoms together with motor weakness) and brainstem aura (at least two of dysarthria, vertigo, tinnitus, hyperacusis, diplopia, ataxia and a decreased level of consciousness).

Data was also collected on confusional migraine aura, defined as disorientation and anterograde amnesia, with or without a decreased level of consciousness, [19] preceding the headache and not requiring hospital admission.

CADASIL encephalopathy was diagnosed clinically as an acute reversible encephalopathy with evidence of reduced consciousness in the absence of any other organic cause, where symptoms lasted for longer than 24 hours,[17] and were sufficient to warrant hospital admission.

Stroke was defined as a clinical stroke syndrome with MRI confirmation of a subcortical lacunar infarct at a site corresponding anatomically with the symptoms. Haemorrhagic strokes or cortical infarcts were excluded from analyses.

Patients were asked about their responses to migraine treatment, and these were divided into two categories: (a) response: a consistent or partial reduction in severity or frequency of migraine attacks; or (b) no response: no response or worsening of migraine attacks. Prophylactic therapy was defined as medication taken specifically for the prevention of migraines, and not in response to an acute attack.

All patients have given written informed consent and the study was approved by the South Thames Research Ethics Committee.

### Statistical methodology

Quantile–quantile plots and the Shapiro-Wilk test were used to estimate distributions of ages at onset of migraine, encephalopathy and stroke. Where distributions of ages at onset were non-normal, the Mann-Whitney U test was used to calculate differences between groups. Odds ratios were calculated using the 2x2 Fisher’s exact test, or a logistic regression analysis where there were multiple categorical variables. Statistical analyses were performed using the R statistical software (version 3.2.2). Probability values of p<0.05 were considered statistically significant.

To assess the association between migraine and lacunar stroke risk a competing risks regression analysis was used to estimate the difference in cumulative incidence of lacunar stroke between migraineurs and non-migraineurs and between the sexes. In competing risks analyses, patients are subject to multiple outcomes (‘failures’), one of which may preclude the other, such as stroke (in this case, the outcome of interest) and death (a competing risk). A competing risks regression analysis can be used to demonstrate the cause-specific hazard of an event, as well as the cumulative incidence of the event while taking into account the influence of competing risks. It is thus superior to conventional survival analyses, which assume that competing events do not occur.

## Results

### The pattern of migraine in CADASIL

**Fig 1:**
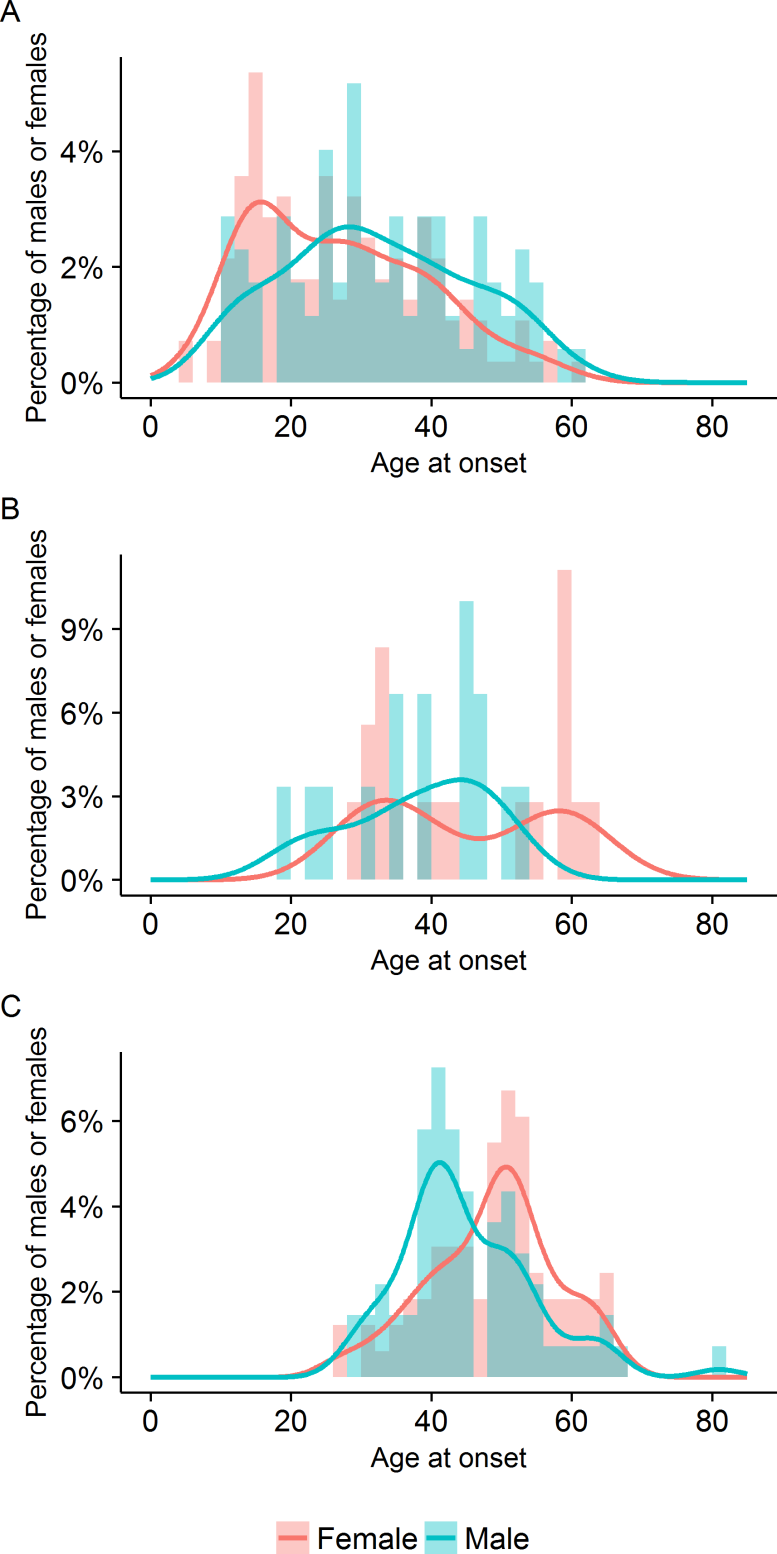
Distribution of ages at onset of (A) migraine, (B) first episode of encephalopathy and (C) first stroke.

226 of 300 cases (75.3%) had a history of migraine. Migraine was the first feature of CADASIL in 203 (67.7%) (Fig 1). Migraine was more common among females (139/170, 81.8%) than males (87/130, 66.9%).

**Fig 2:**
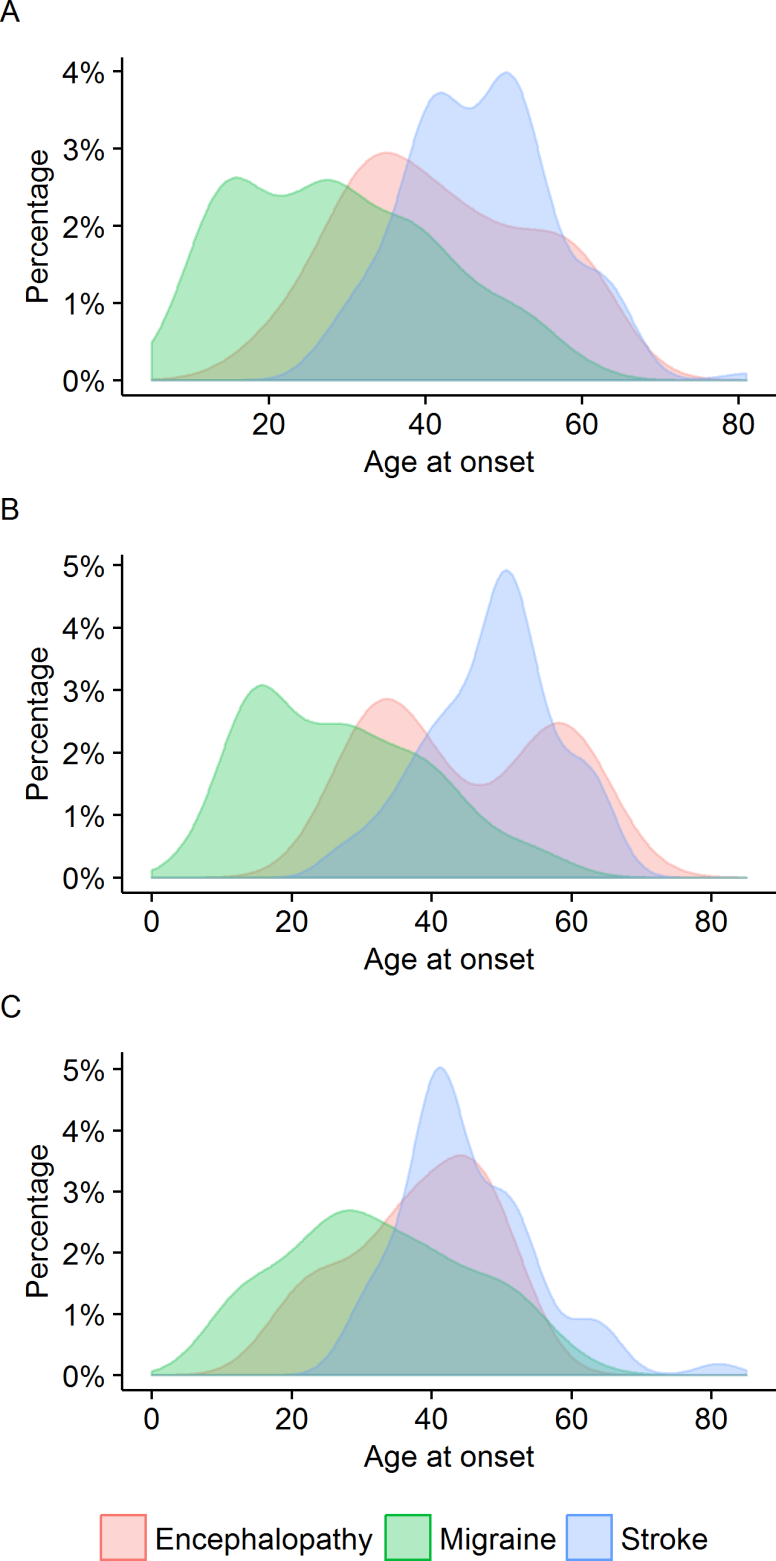
**Ages at onset of migraine, encephalopathy and stroke for (A) all symptomatic patients, (B) females and (C) males.** Migraine was often the first feature of CADASIL, and preceded 75.8% of encephalopathic episodes.

The age at onset of migraine was highly variable, with a median of 28 years (interquartile range 20, range 5–61, mean ± SD 29.0 ± 13.1). The age at onset was earlier in females (median 25 years, interquartile range 20.5, mean ± SD 26.9 ± 12.7) than in males (median 31 years, interquartile range 18, mean ± SD 32.3 ± 13.2) (p = 0.004, w = 7422) and followed a left-skewed distribution in both sexes (Table 1, Fig 2).

**Table 1:** Features of migraine and stroke in 300 symptomatic CADASIL patients.

		Male	Female	Total	p-values
**All symptomatic patients**		130	170	300	
**Migraine**		87 (66.9%)	139 (81.8%)	226 (75.3%)	
**Migraine as first feature**		75 (57.7%)	128 (75.3%)	203 (67.7%)	
**Migraine age at onset (years)**	Median (interquartile range)	31 (18)	25 (20.5)	28 (20)	Mann-Whitney U test: Males vs. females: p = 0.004, w = 7422
	Mean (SD)	32.3 (13.2)	26.9 (12.7)	29.0 (13.1)	
	Range	10–61	5–60	5–61	
**Migraine without aura**		9 (6.9%)	19 (11.2%)	28 (9.3%)	
**Migraine with aura**		81 (62.3%)	122 (71.7%)	203 (67.7%)	
**Migraine with and without aura**		3 (2.3%)	2 (1.2%)	5 (1.7%)	
**Migraine with aura (Percentage of those with aura)**	Typical aura	80 (98.8%)	119 (97.5%)	199 (98.0%)	
	Typical aura with headache	75 (92.5%)	111 (91.0%)	186 (91.6%)	
	Typical aura without headache	10 (12.3%)	19 (15.6%)	29 (14.3%)	
	Hemiplegic migraine	14 (17.3%)	19 (15.6%)	33 (16.3%)	
	Confusional aura	17 (21.0%)	23 (18.9%)	40 (19.7%)	
Number of auras* **experienced (Percentage of those with aura)**	1	51 (63.0%)	83 (68.0%)	134 (66.0%)	
	2	29 (35.8%)	36 (29.5%)	65 (32.0%)	
	3	1(1.2%)	3(2.5%)	4 (1.9%)	
**Stroke (all)** ^†^		69 (53.1%)	82(48.2%)	151 (50.3%)	
	Lacunar stroke	68 (52.3%)	81 (47.6%)	149 (49.7%)	
	Recurrent strokes (percentage of patients with lacunar stroke)	34 (50.0%)	30 (37.0%)	64 (43.0%)	
**Lacunar stroke age at onset (years)**	Median (interquartile range)	43 (11)	50 (11)	48 (13)	Mann-Whitney U test: Males vs. females: p = 0.003, w = 1976.5
	Mean (S.D.)	44.9 (9.6)	48.8 (9.2)	47.0 (9.5)	
	Range	28–81	26–67	26–81	
**Lacunar stroke–past medical history of migraine (Percentage of those with lacunar stroke)**		26 (38.2%)	57 (70.4%)	83 (55.7%)	

CADASIL patients diagnosed on pre-symptomatic genetic testing were not included in this study. Migraine was classified according to the ICHD-3 beta, with aura classified according to typical aura (visual, sensory and/or speech or language symptoms and no motor weakness or monocular field defect) and hemiplegic migraine (visual, sensory and/or speech or language symptoms, as well as motor weakness). Data was also collected for patients who experienced confusional aura.

*Aura types include typical, hemiplegic or confusional aura.

^†^All strokes were subcortical lacunar infarcts, apart from one case of fatal brainstem haemorrhage in a patient on warfarin, and one patient with a cerebellar vermis haemorrhage. Haemorrhagic strokes were not included in subsequent analysis.

203 (67.7%) patients with migraine experienced aura, with the majority of patients reporting more than one type of aura (Table 1). Of these 203 patients, 199 (98.0%) experienced typical aura, 33 (16.3%) fulfilled criteria for hemiplegic migraine, and 42 (20.7%) reported confusional aura (Table 1). None of these patients reporting confusional aura fulfilled criteria for brainstem aura.

### Responses to therapies

A variety of acute and preventive drugs had been used in treatment of the migraine. Of 226 patients with migraine, information on the treatment of migraine was available in 213 (94.2%). 103 (48.4%) were taking at least one medication for their migraine. 74 (34.7%) had taken medication for acute relief of migraine, and 48 (22.5%) had taken medication as migraine prophylaxis at some time. The treatments used and their responses are shown in Table 2.

**Table 2:** Treatment and responses to acute and prophylactic management of migraine in CADASIL.

Drug		No. of patients (n = 213)	Response	No response	Effect unknown
**No drugs used in migraine treatment**		110 (51.6%)	-	-	-
**Drugs used in migraine treatment**		103 (48.4%)			
	**For prophylaxis**	48 (22.5%)	31	10	7
	**For acute relief**	74 (34.7%)	43	14	17
**Drugs used for acute treatment of migraine**					
Simple analgesics*		31	20	4	7
	**Paracetamol**^**†**^	11	9	1	1
	**Ibuprofen**	8	5	2	1
	**Aspirin**^**†**^	8	7	0	1
	**(Unspecified)**	6	1	1	4
**Paracetamol and codeine**		30	20	5	5
	**Co-codamol**	3	1	1	1
	**Co-dydramol**	2	1	0	1
	**Migraleve**^**†**^	25	18	4	3
**Co-proxamol (paracetamol + dextropropoxyphene)**		1	1	0	0
**Other NSAIDs**	**Indomethacin**	1	1	0	0
**Other opioid analgesics**		3	2	0	1
	**Tramadol**	1	0	0	1
	**Morphine**	2	2	0	0
Triptans*^†^		24	10	12	2
	**Sumatriptan**^**§**^	18	9	9	1
	**Naratriptan**	2	0	1	1
	**Zolmitriptan**	3	1	2	0
	**Lyophilisate rizatriptan**	2	0	2	0
	**(Unspecified)**	2	1	1	0
**Primidone**		1	0	1	0
**Domperidone**		1	1	0	0
**Diazepam**		1	0	1	0
**Drugs used as regular migraine prophylaxis**					
β blockers*		25	10	8	10
	**Propanolol**^**†**^^**‡**^	22	8	8	6
	**Atenolol**	1	0	1	0
	**Metoprolol**	1	0	0	1
	**(Unspecified)**	2	1	0	1
**Calcium channel blockers**		4	3	1	0
	**Flunarizine**	1	1	0	0
	**Verapamil**	2	1	1	0
	**Nimodipine**	1	1	0	0
**Pizotifen**^**‡**^		16	8	5	3
**Gabapentin**		4	2	2	0
**Amitriptyline**^**‡**^		15	6	5	5
**Anticonvulsants**		6	4	2	0
	**Topiramate**	3	2	1	0
	**Sodium valproate**	3	2	1	0
**Ergolines**		2	0	1	1
	**Methysergide**	1	0	0	1
	**(Unspecified)**	1	0	1	0
**Acetazolamide**		1	0	0	1
**Fluoxetine**		1	0	1	0
**Combinations**	**Pizotifen + Amitriptyline**	2	2	0	0
	**Amitriptyline + Propranolol**	1	1	0	0
	**Pizotifen + Amitriptyline + Propranolol**	1	1	0	0

*Some patients have tried more than one type of drug in this class.

^†^Drugs used both as regular migraine prophylaxis and pro re nata in acute treatment.

^**‡**^Drugs which failed in isolation or in some combinations but showed good responses in other combinations.

^§^Drugs with responses that later wore off.

For acute management, simple analgesics such as paracetamol, ibuprofen and aspirin were most commonly used (in 31 of 74 patients, 41.8%), and were reported to have a good or partial response in 20 patients, (64.5%). A combination of paracetamol and codeine (the most common formulation used was Migraleve) had been used in 30 patients (40.5%), with a good or partial response in 20 patients (66.7%).

Twenty four patients had taken triptans with data on responses available in 22; of these they were associated with a response in 10 (45.5%) and lack of response in 12 (54.5%). There were no strokes or worsening of neurological status reported on triptans (Table 2). One patient reported that the effectiveness of sumatriptan wore off after repeated use.

The most common drugs used for migraine prophylaxis were beta-blockers. With these 10 out of 25 (32%) patients reported a response (40%) and 8 (32%) reported no benefit. Responses to other prophylactic agents are shown in Table 2.

### The relationship between migraine and encephalopathy

33 (11.0%) patients had a history of CADASIL coma or encephalopathy and this was the presenting feature leading to a diagnosis of CADASIL in 26 (8.7%), and the first feature of CADASIL in 2 (0.67%) (Fig 2). Often initially diagnosed as a viral encephalitis, these episodes recurred in 10 (30.3%) patients. The age at onset of encephalopathy followed a bimodal distribution in females and a right-skewed distribution in males (Fig 1B). The median age at onset of encephalopathy was 40 years (interquartile range 19, mean ± SD 41.8 ± 12.1, and range 19–63). The age at onset of encephalopathy did not differ between the sexes (p = 1.0) (Table 3). Four patients developed encephalopathy during the perinatal or postpartum period.

**Table 3:** Features of CADASIL coma or encephalopathy in 33 patients with CADASIL.

Feature		Male	Female	Total	p-values
**Encephalopathy (%)**		15 (11.7%)	18 (10.8%)	33 (11.0%)	
	**Encephalopathy as a presenting feature**	11 (8.5%)	15 (8.8%)	26 (8.7%)	
	**Encephalopathy as first feature**	2 (1.5%)	0	2 (0.67%)	
**Age at onset of encephalopathy (years)**	**Median (interquartile range)**	38 (13)	41 (25.8)	40 (19)	**Mann-Whitney U test: Males vs. Females**: p = 0.2, w = 100.5
	**Mean (SD)**	38.1 (10.2)	44.9 (12.9)	41.8 (12.1)	
	**Range**	19–52	29–63	19–63	
**Encephalopathy–past medical history of migraine**		13 (13 with aura)	18 (17 with aura)	31 (30 with aura)	
**Duration of encephalopathy (days)**	**Median (interquartile range)**	7 (4.5)	8 (4.8)	8 (5)	**Mann-Whitney U test: Males vs. Females**: p = 1.0, w = 136.5
	**Mean (SD)**	8.2 (3.8)	7.9 (3.3)	8.1 (3.4)	
	**Range**	3–17	3–14	3–17	
**Recurrence of encephalopathy**		4	8	12 (36%)	
**Associated features of encephalopathy**	**Preceded by migraine**	13	12	25 (75.8%)	
	**Fever**	2	2	4 (12.1%)	
	**Hallucinations**	5	8	13 (39.4%)	
	**Meningism**	0	1	1 (0.03%)	
	**Vomiting**	6	3	9 (27.3%)	
	**Seizures**	3	7	10 (30.3%)	

Encephalopathic episodes were characterised by a reduced conscious level, or confusion, lasting up to 3 to 17 days, with a median of 8 days (interquartile range 5). In 25 patients (75.8%) the coma evolved from an acute migraine headache. Common accompanying features were seizures (n = 10), fever (n = 4), hallucinations (n = 13), nausea and/or vomiting (n = 9) and meningism (n = 2) (Table 3). Patients also reported associated focal neurological symptoms typical of migraine aura, such as speech and language disturbances or sensorimotor deficit. Although the majority of encephalopathic episodes resolved completely, five patients reported the persistence of associated symptoms lasting at least 6 weeks after the acute episode.

Data on brain imaging (with diffusion-weighted MRI) during the episodes was available in 8 patients, and there were no acute DWI positive lesions. Cerebrospinal fluid (CSF) analysis results were available in 7 patients, of which 6 showed raised protein levels and two showed leucocytosis. All seven had normal glucose levels. Electroencephalograms (EEGs) were performed in five patients and showed generalized slowing.

Patients with a past history of migraine with aura had a higher odds ratio of developing encephalopathy (30/33 vs. 173/267; OR = 5.4, 95% CI 1.6–28.4, p = 0.002). Migraine sufferers with a history of confusional aura also had an increased risk of developing encephalopathy (OR = 2.5, 95% CI 1.0–5.8, p = 0.04), but there was no increase in risk with other aura types, or with sex or age at onset of migraine.

### Relationship between migraine and lacunar stroke

151 patients (82 females, 48.2% and 69 males, 53.1%) had experienced stroke, all of which were subcortical lacunar infarcts, apart from one case of fatal brainstem haemorrhage in a patient on warfarin, and one patient with a cerebellar vermis haemorrhage, both of which were excluded from subsequent analyses. None of the lacunar infarcts were fatal. 64 patients suffered more than one stroke.

The distribution of ages at onset of lacunar stroke followed a symmetrical and unimodal distribution in both sexes, with a median age at onset of first stroke of 48 years (interquartile range 13, mean 47.0, SD 9.5, range 26–81). The age at onset was higher in females (median 50 years, interquartile range 11, mean ± SD 48.8 ± 9.2) than in males (median 43 years, interquartile range 11, mean ± SD 44.9 ± 9.6), (p = 0.003, w = 1976.5) (Fig 1C, Table 1).

**Fig 3:**
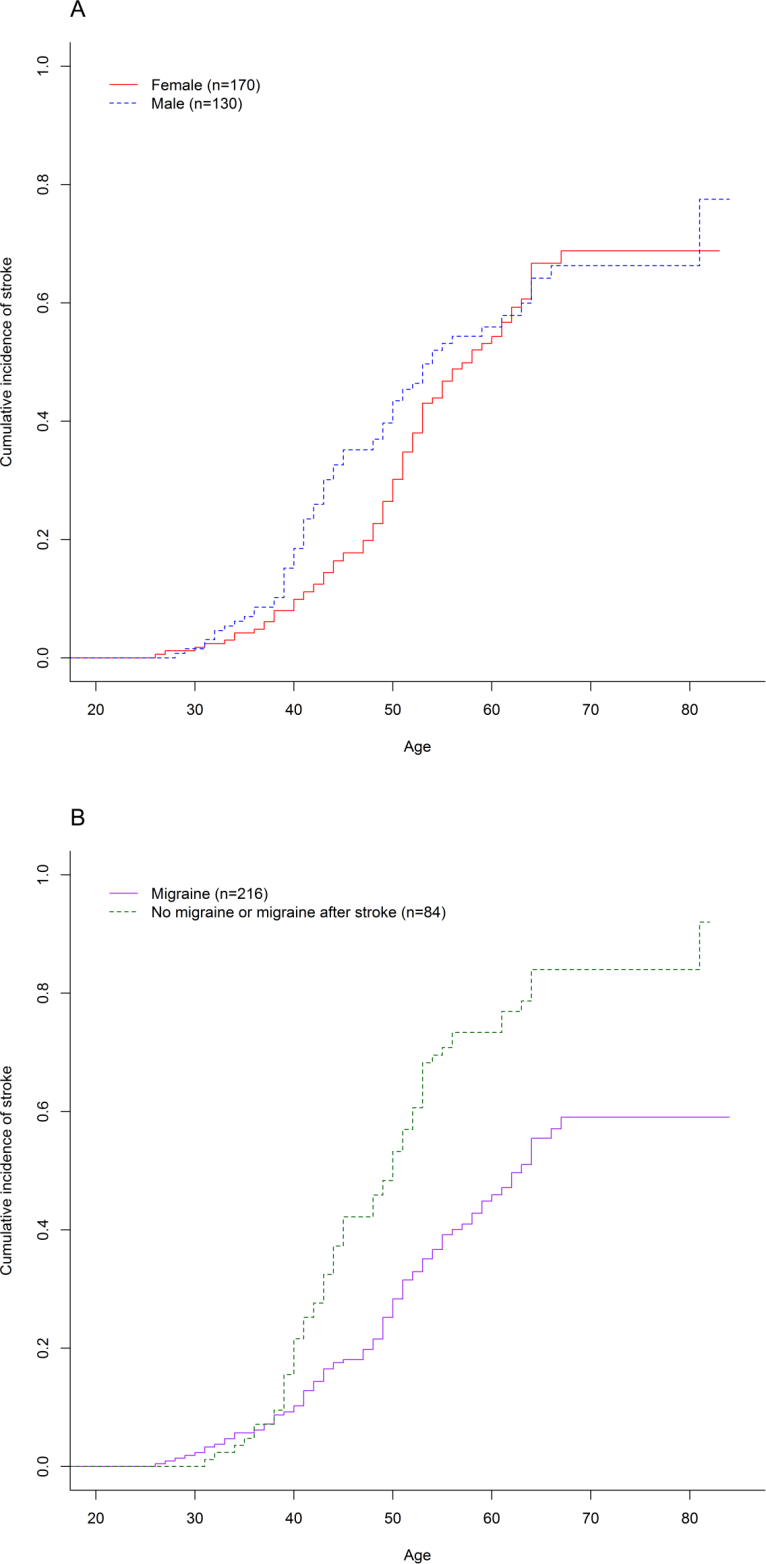
**Cumulative incidence of stroke in (A) males and females, and (B) migraineurs and non-migraineurs**. As demonstrated on competing risks analysis, migraineurs had a lower hazard ratio than non-migraineurs (HR = 0.5, 95% CI 0.3–0.6, p = 2.1 x10^-6^), while the hazard ratio for female sex was not significant (HR = 0.9, 95% CI 0.7–1.3, p = 0.57).

83 (55.7%) patients (57 females, 70.4% and 26 males, 38.2%) had a past medical history of migraine before their first stroke. A history of migraine prior to stroke onset was associated with a lower cumulative incidence of stroke, compared to individuals without migraine or who developed migraine only after their first stroke. On competing risks analysis of the time to first stroke, the hazard ratio for the presence of a history of migraine prior to stroke was 0.5 (95% CI 0.3–0.6, p = 2.1x 10^−6^) (Fig 3). Despite the difference in the ages at onset of stroke between the sexes, the hazard ratio for sex was not significant (HR for females: 0.9, 95% CI = 0.7–1.3, p = 0.57) (Fig 2A).

## Discussion

In this study in 300 symptomatic CADASIL patients, migraine was the presenting symptom in two thirds, and present in three quarters. The nature of migraine differed from that in the general population, with 90% of CADASIL patients experiencing migraine with aura, and prolonged and complex auras being common. This is in contrast to the predominance of migraine without aura in the general population.[20–22] The age at onset of migraine in CADASIL was also later than in the general population.[23] However, consistent with migraine in the general population,[24] as well as previous studies of European CADASIL patients,[25] migraine in CADASIL was more common in females, with a mean age of onset around 4 years earlier.

There is little published data on treatment responses to migraine in CADASIL.[9–12] We found a significant proportion of patients with migraine did not take medication due to the migraine episodes being infrequent. However in those that did, treatment responses seemed to be similar to that seen in the general population.[26,27] Of interest, 24 patients had taken triptans and none had experienced serious side effects. Drug information sheets tend to indicate that triptans are contraindicated in patients with stroke or TIA, or at high risk of cardiovascular disease, because of the theoretical risk that they might exacerbate cerebral ischaemia,[28] but we found no evidence that this occurred. This is consistent with data from the general population which found no association of triptans with stroke risk.[29] A fifth of CADASIL migraineurs required regular prophylactic treatment for migraines, with a variable response to these preventive medications.

Encephalopathy in CADASIL was first described as being completely reversible episodes of confusion, coma, fever and seizures.[17,30] In this study we found that 11% of patients developed such episodes warranting hospitalization. Encephalopathy was also the presenting feature leading to a diagnosis of CADASIL in majority of these 33 patients, hence CADASIL should be considered in the differential of a patient presenting with encephalopathy with inconclusive results on imaging, EEG and CSF analysis. Four patients experienced encephalopathic episodes around the puerperium as previously reported.[31,32] The predilection for the puerperium suggests a possible hormonal contribution, which might also contribute to the increased prevalence of migraine in females.

Encephalopathy episodes have been previously reported to often develop from a migraine attack, and we hypothesised that they might share an underlying aetiology. Consistent with this, 76% evolved from a migraine attack, and we found that encephalopathy was more common in CADASIL cases who had previously experienced migraine with aura. Furthermore, individuals with migraine with aura who had experienced confusional migraine aura were more likely to develop encephalopathy, compared with those with other aura types. Our data is consistent with a continuum of symptoms in CADASIL, ranging from acute confusional migraine episodes lasting up to three days,[8,19] status migranosus with persisting aura lasting up to 8 days,[8] and encephalopathic episodes lasting up to 14 days.[2,17]

Migraine with aura is an independent risk factor for stroke, and is associated with white matter changes and silent infarct-like lesions on MRI.[14] Migraine and stroke also co-occur in other monogenic forms of small vessel disease. We hypothesised that migraine might also contribute to a more severe phenotype in CADASIL; however we found no evidence of this and indeed non-migraineurs with CADASIL had a higher cumulative incidence of stroke. The explanation for this association is uncertain and the finding needs replicating in other cohorts, but it is reassuring that migraine with aura is not associated with a worse phenotype. The electrophysiological basis of migraine in CADASIL is believed to be Cortical Spreading Depression (CSD) in which waves of synchronised depolarisation spread across the brain cortex,[33] being associated first with an increase in cortical blood flow, followed by a period of hypoperfusion.[34] It is possible that such episodes could be associated with protection against subsequent ischaemia, perhaps by mechanisms related to ischaemic preconditioning.[35]

While this is one of the largest studies of migraine, encephalopathy and stroke in CADASIL, it is not without limitations. As this was a cross-sectional analysis of prospectively collected data, it is probable that more severe cases of CADASIL are detected earlier, while there may be many *NOTCH3* mutation-carrying individuals with few or no symptoms that remain undiagnosed. There is currently no clinically accepted definition of encephalopathy in CADASIL. The distinction between complicated migraine with confusional aura and an encephalopathic episode may be difficult–in this study we have set an arbitrary measure of severity (the need for hospitalisation) and a temporal definition (encephalopathic symptoms lasting at least 24 hours).

In conclusion our results provide data from a large population of prospectively recruited CADASIL patients on the frequency, characteristics and treatment responses of migraine. Our data shows that while many patients do not need treatment for their migraine, about half do, and in this group similar treatment responses are seen to that in the general population with migraine. In particular we found no ischemic complications in those taking triptans. Our series further characterises the CADASIL coma or acute reversible encephalopathy which may lie on the spectrum of migraine auras, sharing similar features with confusional migraine episodes.

## Supporting Information

S1 File (XLSX)
